# Pancastatin A and B Have Selective Cytotoxicity on Glucose-Deprived PANC-1 Human Pancreatic Cancer Cells

**DOI:** 10.4014/jmb.2002.02002

**Published:** 2020-04-09

**Authors:** Hae-Ryong Park

**Affiliations:** School of Bioconvergence, Kyungnam University, Changwon 51767, Republic of Korea

**Keywords:** Pancastatin A, pancastatin B, glucose deprivation, GRP78, pancreatic cancer

## Abstract

Glucose deprivation and hypoxia frequently occur in solid tumor cells, including pancreatic cancer cells. Glucose deprivation activates the unfolded protein response (UPR) and causes the upregulation of glucose-regulated protein 78 (GRP78). Induction of GRP78 has been shown to protect cancer cells. Therefore, shutting down of GRP78 expression may be a novel strategy in anticancer drug development. Based on this understanding, a screening system established for anticancer agents that exhibit selective cytotoxicity on pancreatic cancer cells under glucose-deprived conditions. To test this hypothesis, the new compounds isolated, pancastatin A (PST-A) and B (PSTB), from Ponciri Fructus. PST-A and B were identified as glabretal triterpenoid moieties by electrospray ionization mass spectrometry and nuclear magnetic resonance spectroscopic methods. PST-A and B suppressed the accumulation of the UPR hallmark gene, GRP78, during glucose deprivation. Furthermore, PST-A and B showed selective cytotoxicity on PANC-1 pancreatic cancer cells under glucose deprivation. Interestingly, PST-A and B had no effect on these cells under normal growth conditions. Our results suggest that PST-A and B act as novel therapeutic agents to induce selective cell death in glucose-deprived pancreatic cancer cells.

## Introduction

Pancreatic cancer is one of the most serious, aggressive, and deadliest form of malignancies. Ductal adenocarcinoma of the pancreas, which account for 90% of all human pancreatic cancers, are an extremely lethal disease with an overall 5-year survival rate of approximately 5% [1-3]. Pancreatic cancer is characterized by its resistance to almost all standard chemotherapy regimens due to the tolerance of cells to apoptosis. Therefore, there is a need to develop effective novel anticancer agents that have specific activity against pancreatic cancer cells. Hence, I focused on singularity of pancreatic cancer, a representative solid tumor, to establish a molecular target for the development of selective chemotherapeutic drugs [4-6].

Pancreatic cancer cells are characterized by their survival in an extreme environment of glucose deprivation, hypoxia, and nutrient deprivation [7, 8]. Glucose deprivation is a physiological stress condition that is induced in several human diseases, such as ischemia and cancer [9]. This stress condition leads to the accumulation of unfolded or misfolded proteins in the lumen of endoplasmic reticulum (ER) in eukaryotic cells [10, 11]. The accumulation of unfolded proteins in the ER activates the unfolded protein response (UPR), which enhances cell survival by limiting the accumulation of unfolded or misfolded proteins in the ER [12]. Tumor cells need to adapt to the severe microenvironmental conditions, and induction of UPR is an important system in this response. UPR can be induced by glucose deprivation and the expression of glucose-regulated protein 78 (GRP78, also known as BiP) [12]. GRP78, also referred to as the immunoglobulin heavy chain binding protein, has been demonstrated to play a role in protecting tumor cells against intracellular-mediated cytotoxicity and against the toxic effects of anticancer agents [13, 14]. GRP78 binds to the luminal domains of IRE1, ATF6, and PERK to maintain them within the ER, and activates these signaling pathways [15, 16]. Upregulation of GRP78 expression in solid tumors protects tumor cells against various stresses. Thus, substances that directly down-regulate GRP78 expression might potentially be of use in the treatment of solid tumors, which are averse to successful cancer chemotherapy.

Based on this understanding, a novel screening system established to discover anticancer agents that process selective cytotoxicity against human pancreatic cancer cells under glucose deprivation. I recently screened 440 herbal medicines, and ensuring their stability, isolated two active compounds ensured stability, JS311-1 and 2, from Ponciri Fructus. By examining their structures and physicochemical properties, they were identified to possess glabretal triterpenoid moieties, and have not been previously reported as new compounds. These new compounds were designated as pancastatin A (PST-A) and B (PST-B).

In this study was investigated the effect of PST-A and B on GRP78 expression in pancreatic cancer cells exposed to glucose deprivation. Furthermore, it demonstrated the selective cytotoxicity of PST-A and B towards glucose-deprived PANC-1 human pancreatic cancer cells.

## Materials and Methods

### Isolation and Purification of Pancastatin A (PST-A) and B (PST-B)

**Fig. 1:**
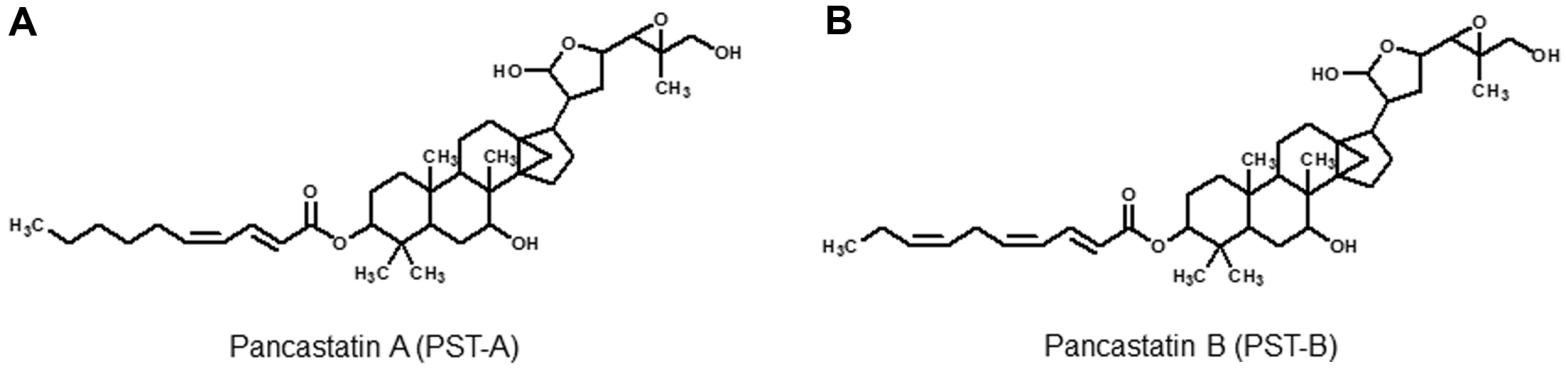
Chemical structures of Pancastatin A (PST-A) and B (PST-B).

Ponciri Fructus was obtained from Kumkang Pharm Co. Ltd., Changwon, South Korea. The dried Ponciri Fructus (1.8 kg) was extracted using 20 L of methanol for three days at room temperature and filtered through Whatman filter paper No. 1 (Advantec, Japan). Then, the methanol was removed by evaporation *in vacuo*, and a dried methanol extract was obtained. The resulting aqueous solution was extracted with ethyl acetate. The active material was applied to a silica gel column and eluted using CHCl_3_:CH_3_OH (100:1 to 1:1 [v:v]). Each fractions was subjected to analysis using thin layer chromatography (TLC). Selective cytotoxic activity of each fraction against the PANC-1 cells was then evaluated, and 50:1 fraction was found to be the most effective in inducing selective cytotoxicity under glucose-deprived conditions. The 50:1 fraction was applied on a Sephadex LH-20 column and eluted with CH_3_OH. The active components that were eluted were purified by ODS sep-pak cartridge, using 80%CH_3_OH. To separate the major compound, high performance liquid chromatography (HITACHI L-2130, Japan) was performed in an ODS column with CH_3_OH-H_2_O (85:15 [v/v]) as the eluent. Active compounds were obtained as a single peak detected at 267 nm. Repeated HPLC finally gave two active compounds, JS-311-1 and JS-311-2. The structures of JS311-1 and 2 were determined using electrospray ionization mass spectrometry (Shimadzu LCMS-IT-TOF, Japan) and nuclear magnetic resonance (JEOL JNM-ECA600) spectroscopic methods. From the comparison of database and literature searches, we found that JS311-1 and 2 were novel compounds, and JS311-1 and 2 were designated as pancastatin A (PST-A) and B (PST-B), respectively (Fig. 1) [17].

### Cell Culture and Treatments

PANC-1 human pancreatic adenocarcinoma cells were obtained from the Korea Cell Line Bank (Republic of Korea). The cells were maintained in DMEM medium (Gibco BRL, USA) supplemented with 10% heat-incubated fetal bovine serum (FBS; Hyclone, USA), penicillin (100 U/ml), streptomycin (100 μg/ml) and 3.7 mg/ml NaHCO_3_. PANC-1 cells were cultured in a 37°C in humidified atmosphere containing 5% CO_2_. To induce glucose deprivation, added a chemical stress, 2-deoxyglucose (2DG). 2DG as a chemical stressor is a substance such as hypoglycemia-mimicking agent [11, 12]. 2DG was purchased from Sigma (USA) and dissolved in sterilized distilled water at a stock concentration of 2 M. 2DG was added to the culture medium at a final concentration of 20 mM.

### Observation of Morphological Changes

PANC-1 cells in DMEM medium containing 10% FBS were seeded in 6-well plates (2.0 × 10^5^ cells/ml) and incubated at 37oC with 5% CO_2_ for 48 h. The cells were treated with various concentrations of PST-A and B. After 30 min incubation, 2DG was added to the well with a final concentration of 20 mM, and the plates were reincubated. After incubation for 24 h, the cellular morphology was observed using an inverted microscope (Nikon, Japan) at 100 × magnification.

### Colony Formation Assay

PANC-1 cells were seeded at 1 × 10^5^ cells/ml in 24-well plates and, incubated for 48 h in a humidified atmosphere of 5% CO_2_ at 37°C. Cells were pretreated with various concentrations of PST-A and B. After 30 min incubation, 2DG was added to the wells a final concentration of 20 mM, and the plates were reincubated for 24 h. For the colony formation assay, the cells were then diluted in new medium, replated at 1.0 × 10^3^ cells/ml per well in 6-well plates, and cultured for 7 to 8 days at 37oC in a humidified atmosphere containing 5% CO_2_. Formed colonies were fixed with 10% formaldehyde, stained with 0.01% crystal violet, and counted. Cell survival (mean values with 95% confidence intervals from triplicate determinations) was calculated by setting the survival of control cells as 100%. IC_50_ values (concentration required for 50% inhibition of colony formation) were determined from the dose-response curves of colony formation inhibition.

### Western Blotting Analysis

Whole cell lysates were prepared by solubilizing cell in 1×sodium dodecyl sulfate (SDS) sample buffer (62.5 mM Tris-HCl, pH 6.8, 2% SDS, 5% 2-mercaptoethanol, and 10% glycerol) as described. Equal amounts of proteins were resolved on 4~20% gel using SDS-PAGE and transferred onto an immunoblot PVDF membrane (Bio-Rad, USA) for western blotting. Nitrocellulose membranes containing the transferred proteins were blocked in Tris-buffered saline containing 5% non-fat dry skim milk and 0.1% Tween 20 for 1 h at room temperature. Membranes were probed with anti-KDEL mouse monoclonal antibody (for detection of GRP78; StressGen, Victoria, British Columbia, Canada) at 1:500 and anti-β-Actin mouse monoclonal antibody (Sigma) at 1:1000. Anti-mouse IgG HRP (Amersham Pharmacia Biotech, Japan) at 1:500 was used as a secondary antibody. β-actin protein was used as the loading control for data normalization. The blots were developed using the enhanced chemiluminescence detection kit (Amersham Pharmacia Biotech) according to the manufacturer’s instructions.

### Hoechst Staining

The morphological change characteristic apoptosis were investigated by staining the cells with Hoechst 33342 (Sigma). PANC-1 cells (2 × 10^5^ cells/ml) were then washed with phosphate-buffered saline (PBS) and fixed in PBS containing 10% formaldehyde for 4 h at room temperature. Fixed cells were washed with PBS, and stained with Hoechst 33342 (10 μM) solution at room temperature in the dark for 30 min. The cells were washed twice more with PBS and the hoechst-stained nuclei were visualized by using a fluorescence microscope (TS 100-F; Nikon). Photographs were taken at 400 × magnification.

### Statistical Analysis

All data presented were the means as a standard deviation of three determinations. Data were analyzed using the SPSS package for Windows (Version 14.0; USA) and evaluated by one-way analysis of variance (ANOVA) followed by Scheffe’s test. The differences were considered significant at *p* < 0.05.

## Results

### Selective Cytotoxic Effect of PST-A and B on the Glucose-deprived PANC-1 Cells

**Fig. 2:**
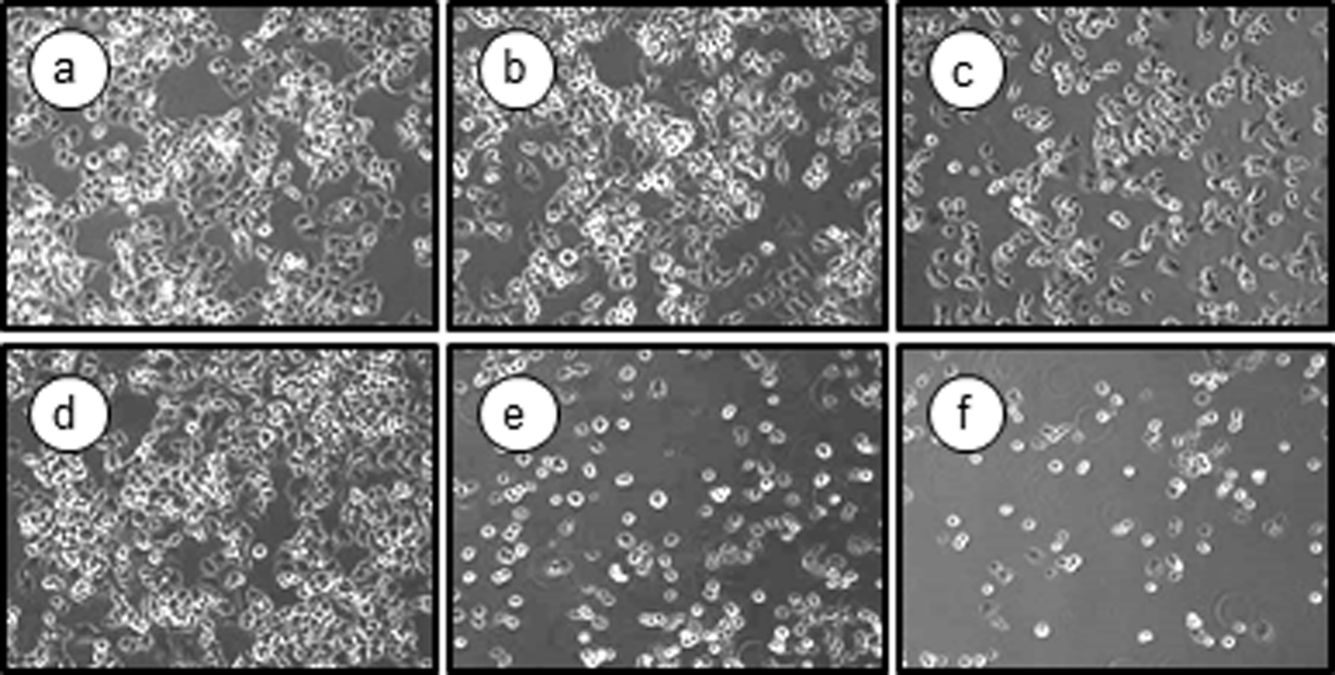
PST-A and B induced morphological changes in glucose deprived PANC-1 cells. The cells were exposed to the indicated 10 μM of PST-A and B under normal and glucose deprivation conditions. PANC-1 cells were exposed to the PSTA and B for 24 h in the present or absent of 2DG. PANC-1 cells indicated highly selective cytotoxicity during 2DG (20 mM) stress conditions (a, normal-control; b, normal-10 μM PST-A; c, normal-10 μM PST-B; d, 2DG-control; e, 2DG-10 μM PST-A; f, 2DG-10 μM PST-B). Photographs were taken at 100× magnification.

To investigate whether PST-A and B have a selective cytotoxicity in glucose-deprived PANC-1 human pancreatic cancer cells, cells were pretreated with various concentrations of PST-A and B in the presence or absence of 2DG. The cytotoxic effects of PST-A and B on PANC-1 morphological alterations were determined by phase-contrast microscopy. Fig. 2 shows PANC-1 cells exposed to 10 μM of PST-A and B under the presence or absence 2DG. PST-A and B exhibited no cytotoxicity under normal growth conditions. However, cells exhibited cytoplasmic shrinkage and were either detached from each other or floated in the medium under 2DG stress condition.

**Fig. 3:**
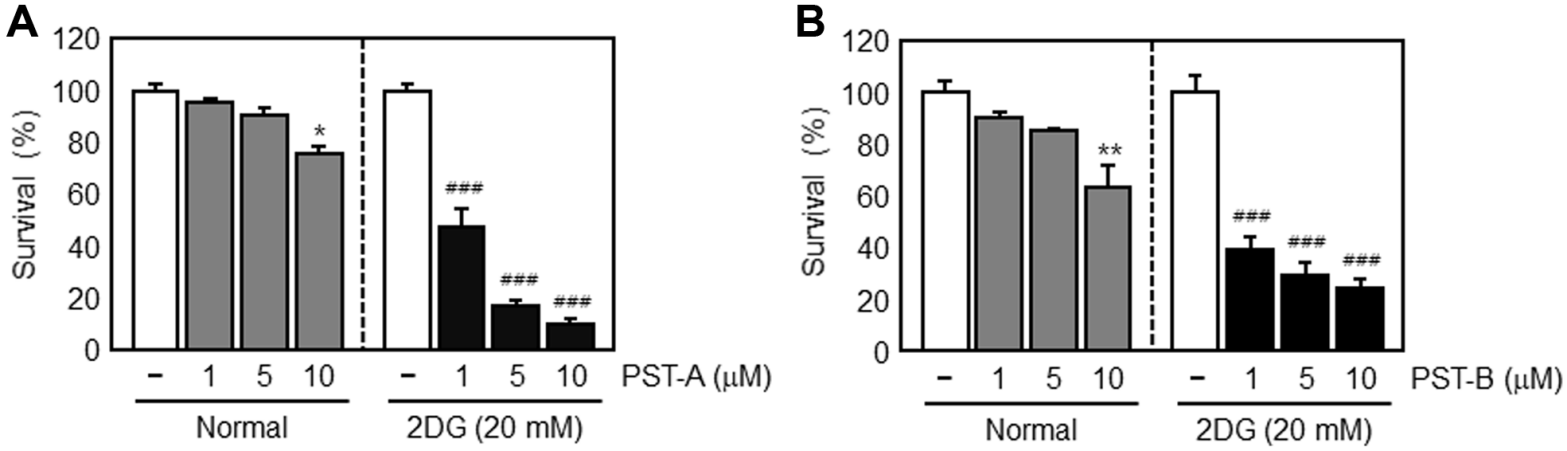
Selective cytotoxicity of PST-A and B under normal and glucose deprivation. Colony formation analysis of PANC-1 cells exposed to the indicated concentration of PST-A and B for 24 h in the presence and the absence of 2DG, the survival rate was calculated by setting each of the control survival rates. After 7-8 days, the formed colonies were fixed with 10% formaldehyde, stained with 0.01% crystal violet, and counted. *p < 0.05; **p < 0.01 compared with normal growth control. ###*p* < 0.001 compared with glucose deprivation control.

We then examined the specific cytotoxic effect of PST-A and B on PANC-1 cells under glucose-deprived conditions by using the colony formation assay. Under normal growth conditions, PST-A and B treatment of PANC-1 cells had only a weak effect on cell viability. In contrast, cells exposed to PST-A and B at 1, 5, and 10 μM showed increase in cytotoxicity as compared to the normal growth controls (Figs. 3A and 3B). Therefore, these results indicate that PST-A and B are not cytotoxic to PANC-1 cells under normal growth conditions. However, PST-A and B treatment led to reduced pancreatic cancer cell viability under glucose deprivation.

### Effect of PST-A and B on GRP78 Protein Expression in Glucose-deprived PANC-1 Cells

Glucose deprivation is a physiological cell condition that leads to accumulation of unfolded or misfolded proteins in the ER lumen of eukaryotic cells. The increase in the unfolded proteins initiates the UPR. The transcriptional activation of GRP78 is the hallmark of UPR that plays a critical role in tumor development, progression, and resistance to chemotherapy [18]. Up-regulation of GRP78 appears to promote tumor growth and progression as well as drug resistance. Agents targeting the UPR pathway may provide a novel therapeutic strategy against cancer. GRP78 is induced during glucose deprivation as part of the UPR, which protects cells from apoptosis under glucose-deprived conditions [19]. Therefore, these results were interested in whether PST-A and B affect GRP78 induction caused by glucose-deprived conditions. Since GPR78 induction constitutes one of the key pro-survival mechanisms of cancer cells in response to glucose deprivation, PST-A and B-induced suppression of GRP78 expression may result in cell death. This assumption was supported by the observed correlation between the suppressed expression of GRP78 by PST-A and B and cytotoxicity.

**Fig. 4:**
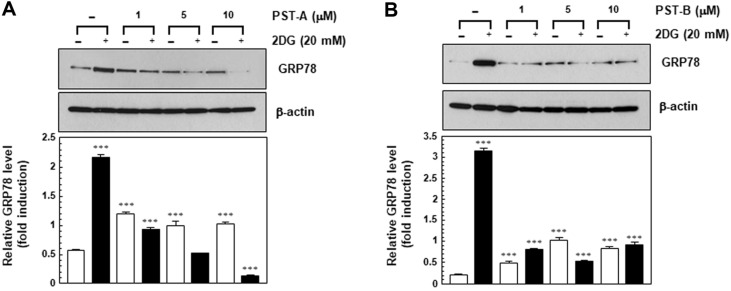
PST-A and B induced the down-regulation of GRP78 in the PANC-1 cells. Total cell lysates of PANC-1 cells were prepared and subjected to western blot analysis using mouse monoclonal anti-KDEL antibody. PNAC-1 cells were treated with PST-A and B indicated concentration for 24 h under 2DG (20 mM) conditions. β-actin was measured as an internal loading control. ***p < 0.001 compared with normal growth control.

To determine the potential GRP78 inhibitory activity of PST-A and B in glucose deprived conditions, the GRP78 protein was performed western blot analysis of the protein samples obtained from PANC-1 cells that had been subjected to glucose deprivation for 24 h in the presence or absence of PST-A and B. PST-A and B treatment resulted in the down-regulation of stress-induced GRP78 protein expression in a dose-dependent manner in the 2DG-treated PANC-1 cells. However, no such effect on the expression level of GRP78 was observed under normal growth conditions. These results provide evidence that PST-A and B suppress the GRP78 expression induced by glucose-deprived conditions as observed by the inhibition of tumor growth in glucose deprived PANC-1 cells (Figs. 4A and 4B). Suppression of GRP78 expression in glucose deprived conditions leads to reduced cell survival and increased apoptosis. Thus, PST-A and B may be good candidate to explore potential cancer therapy.

### PST-A and B Induce the Apoptotic Response in Glucose-deprived PANC-1 Cells

Apoptosis is the mechanism by which cells undergo programmed cell death to balance cell proliferation or respond to DNA damage [20]. It is characterized by membrane blebbing, shrinking of the cytoplasm, nuclear fragmentation, and the formation of distinct apoptotic bodies [21]. Apoptosis plays an important role in maintenance of tissue homeostasis and protects against carcinogenesis by eliminating damaged cells or abnormal excess cells [22, 23]. The understanding of apoptosis provided the basis for targeted therapies, and many chemotherapeutic agents exert their anticancer effects by inducing apoptosis in cancer cells. Therefore, induction of apoptosis is relevant to understanding cancer and developing more effective anticancer therapy [24].

**Fig. 5:**
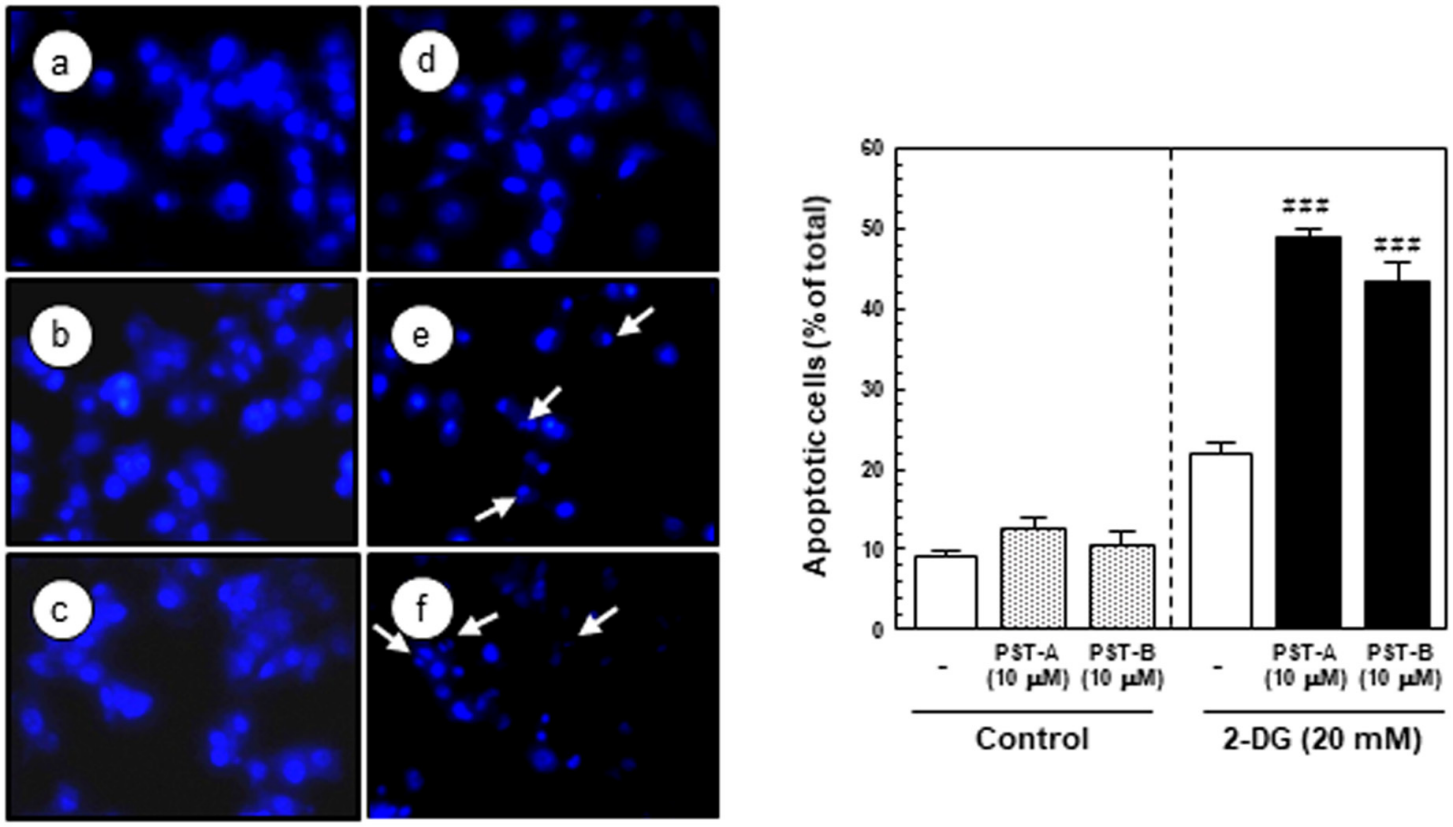
PST-A and B induced apoptosis under 2DG stress conditions. The cells were exposed to the indicated 10 μM of PST-A and B under normal and glucose deprivation condition. PANC-1 cells were exposed to the PST-A and B for 24 h in the present or absent of 2DG. (**A**) Apoptosis nuclear and condensation and fragmentation induced by PST-A and B on contained 2DG PANC-1 cells (a, normal-control; b, normal-10 μM PST-A; c, normal-10 μM PST-B; d, 2DG-control; e, 2DG-10 μM PSTA; f, 2DG-10 μM PST-B). Photographs were taken using a blue filter at a magnification of × 400. Arrow indicates an apoptotic cell with apoptotic bodies. (**B**) This histogram represents the percentage of apoptotic PANC-1 cells in total cell population after different treatment. ###*p* < 0.001 compared with glucose deprivation control.

To investigate the mechanism of the cell death induced by PST-A and B, examined the apoptotic activity of PANC-1 cells in response to PST-A and B treatment under glucose deprivation conditions. I investigated several hallmarks of apoptosis, such as nuclear chromatin condensation and fragmentation of DNA using Hoechst 33342 staining. Apoptotic cells was collected and counted after PST-A and B treatment in the presence or absence of 2DG and examined the morphological changes in their nuclei. Pancreatic cancer cells treated with 10 μM of PST-A and B showed marked chromatin condensation and the formation of apoptotic bodies in the cell group exposed to 2DG, but no such observation was made for the cell group that was not exposed to 2DG (Fig. 5).

## Discussion

Solid tumors, especially pancreatic cancer, are surrounded by stressful microenvironments, including glucose deprivation, hypoxia and nutrient deprivation [25]. These microenvironmental conditions, especially glucose deprivation, activate the UPR, a stress-signaling pathway, in tumor cells [26, 27]. Tumor cells need to adapt to the microenvironmental conditions and induction of UPR is an important system in this response. GRP78 binds to the ER transmembrane sensor proteins and maintains them in an inactive form. The accumulation of unfolded proteins in the ER activates the UPR, which enhances cell survival by induction of GRP78 [28, 29]. Several reports have demonstrated that GRP78 plays a role in protecting tumor cells against the toxic effects of anticancer agents [12-14]. GRP78 has been established to be important for tumor progression and chemotherapeutic drug resistance [30, 31]. Induction of GRP78 expression in solid tumors protects tumor cells against glucose deprivation. Therefore, GRP78 proteins in glucose-deprived cells may be a potent therapeutic target for anticancer therapy [32].

In this study have demonstrated that the PST-A and B have selective cytotoxicity on pancreatic cancer cells under 2DG-induced glucose deprivation. 2DG is a synthetic analogue of glucose in which the hydroxyl group at the second position carbon is replaced by hydrogen, and causes a block in glycolysis and glycosylation [33]. 2DG also induces UPR in the ER due to low glucose stress [34]. I isolated novel compounds from Ponciri Fructus, designated pancastatin A (PST-A) and B (PST-B). Ponciri Fructus has previously been reported to induce anti-inflammatory and anti-helicobacter pylori effect [35]. Specifically, PST-A and B inhibited the expression of the UPR target gene, GRP78 during glucose deprivation. Disruption of the UPR may therefore, provide a novel therapeutic approach to targeting glucose-deprived solid tumors.

This study indicates that PST-A and B can be potential candidates and a novel therapeutic approach to induce selective cell death under glucose deprivation in pancreatic cancer cells. As shown in Figs. 2 and 3, PST-A and B had an inhibitory effect on tumor cell viability of pancreatic cancer cells under glucose-deprived conditions. Expectedly, they had no effect under normal growth conditions. Moreover, as shown in Fig. 5, PST-A and B lead to apoptosis. PANC-1 cells treated with PST-A and B showed chromatin condensation and the formation of apoptotic bodies in the group to 2DG. On the assumption that, verified mechanisms of the PST-A and B connoted these selective killing activities. As expected, western blotting confirmed that PST-A and B significantly decreases the 2DG induced GRP78 protein levels in PANC-1 cells (Fig. 4).

In conclusion, PST-A and B had no cytotoxic effect under normal growth conditions, but showed selective cytotoxicity against PANC-1 cells under glucose deprivation. This study indicated that PST-A and B induces GRP78 down-regulation in glucose deprived PANC-1 pancreatic cancer cells. These results indicate that PST-A and B may be potential novel therapeutic candidates to induce selective cell death under glucose deprivation in pancreatic cancer cells.
